# Supplementary material for: Chromothripsis during telomere crisis is independent of NHEJ, and consistent with a replicative origin
Source: Genome Res. 2019 May;29(5):737–49. doi: 10.1101/gr.240705.118 (PMC6499312; doi:10.1101/gr.240705.118)
Supplement: Supplemental Material [file supp_gr.240705.118_Supplemental_file_1.zip › contigs/annotated_contigs/DB55/contig.2.DB55_length_780_mean_cov_4.5858974359.docx]

**DB55_length_780_mean_cov_4.5858974359**

```
TTCTAAAAATAAAGCAGAGACTAAATTTCAAGAAAATTCTCAATTGTTAGAGGTTACAATCGAGTAATCTATGACCTAATATCTCACAA
 >chr14:81295291-81295575 + E=5e-159
AGTCTGCACGAAACATGACTTTGAGAACAGCTTGGGAACAAAGATATTCAAATGGCCTCCAAAAGGTGCCATAAGAACATTAGTGATAC

AGACAATAAACTTGTTCTAACTTAATAGTTCTTTTATGAAAAATAAAGGAAAATCAGACGTGTCACTAAATCAGAAACAGCTGCAAATT

TAATTAACTATTTTATT|AAAAATAAAAAAAAGTATAAA|TAAAAAAAAGTATAAATAGTAACAGGAGACCTGTGTTACTGTTTATAGT
                                       >chr14:81124349-81124795 + E=2e-244
TTTTTTCTAATGCTATCACCACAGTAAAGTACTTTAGATTGATAATTTAGATCTTCCAGAAACATGAAAAAAATTAAGAGTTAATTGGT

TTTTAATTCACCTAGTACAAGTTTTAAAAAGGTAATTTGTCATTTCTGATCTGACATCGCTTATGGGCATGGCAACTATGGAAGGATTA

TGGTAGGGGATATAGAAAATGATAGGGAGAGGAAATTGGTTTTGAGTCGCCTTAGATTGTACATCAACATTCTTTTGTTGCTGTTGTTT

TCAGGCTTTAATGCCCTATTCACATTTATGCTTGTCAGCAAATTTCCCTTGGCTACATTCAAAAGGCAGCTGTTCATCCACACTCCTCA

ATGCATAAATATATCAAAGCCAATTCTTTGCCTGGGGACAT|TGTTTGTTTGCTAGAAGAATTCCCGCCTT
```
